# Applicability and validation of the Reaction to Tests Scale (RTT) in a sample of Portuguese medical students

**DOI:** 10.1186/s40359-021-00656-w

**Published:** 2021-10-27

**Authors:** Daniela S. M. Pereira, Ana Mónica Pereira, Teresa Costa Castanho, Gabriela A. Silva, Filipe Falcão, Patrício Costa, José Miguel Pêgo

**Affiliations:** 1 Life and Health Sciences Research Institute (ICVS), School of Medicine, University of Minho, Largo do Paço, Campus de Gualtar, 4710-057 Braga, Portugal; 2 ICVS/3B’s, PT Government Associate Laboratory, Largo do Paço, 4700-000 Braga, Portugal; 3 iCognitus4ALL – IT Solutions, 4710-057 Braga, Portugal; 4 NOVA Medical School, New University of Lisbon, Campo dos Mártires da Pátria 130, 1169-056 Lisbon, Portugal; 5 iNOVA4Health, CEDOC, Edifício CEDOC II, Rua Câmara Pestana 6, 1150-082 Lisbon, Portugal

**Keywords:** Confirmatory factor analysis, Exploratory factor analysis, Medical students, Test anxiety

## Abstract

**Background:**

Test anxiety is a crucial factor in determining academic outcomes, and it may lead to poor cognitive performance, academic underachievement, and psychological distress, interfering specifically with their ability to think and perform during tests. The main objective of this study was to explore the applicability and psychometric properties of a Portuguese version of the Reactions to Tests scale (RTT) in a sample of medical students.

**Method:**

A sample of 672 medical students completed the RTT. The sample was randomly split in half to allow for independent Exploratory Factor Analysis (EFA) and to test the best fit model—Confirmatory Factor Analysis (CFA). CFA was used to test both the first-order factor structure (four subscales) and second-order factor structure, in which the four subscales relate to a general factor, Test Anxiety. The internal consistency of the RTT was assessed through Cronbach’s alpha, Composite reliability (CR) and Average Variance Extracted (AVE) for the total scale and each of the four subscales. Convergent validity was evaluated through the correlation between RTT and the State-Trait Anxiety Inventory (STAI-Y).To explore the comparability of measured attributes across subgroups of respondents, measurement invariance was also studied.

**Results:**

Results from exploratory and confirmatory factor analyses showed acceptable fits for the Portuguese RTT version. Concerning internal consistency, results indicate that RTT was found to be reliable to measure test anxiety in this sample. Convergent validity of the RTT with both state and trait anxiety STAI-Y’s subscales was also shown. Moreover, multigroup analyses showed metric invariance across gender and curriculum phase.

**Conclusion:**

Our results suggest that the RTT scale is a valid and reliable instrument for the measurement of test anxiety among Portuguese Medical Students.

**Supplementary Information:**

The online version contains supplementary material available at 10.1186/s40359-021-00656-w.

## Introduction

Much research has been done regarding the role of emotion on performance, with anxiety-usually characterized by sentiments of tension, worry and negative physiological reactions-being the key variable of interest in comprehending the role of emotion in performance [1]. Higher levels of anxiety are often manifested in situations in which we are evaluated. These scenarios are part of our routine, both at the academic and at the professional level, and can arise as anxiety and stress enhancers [1, 2]. Although anxiety can be useful, encouraging learning and motivating students, extreme levels can have health repercussions both at the mental and physical levels [2]. One of the most common anxiety situations reported by students is test anxiety [3]. Regarding educational settings, test anxiety is frequently described by context specific stimuli and academic subject specific reactions, being distinguished from other forms of anxiety through its focus on evaluative circumstances [1]. In an academic context, college students (and particularly, medical students) are no exception to the rule [4]. Test anxiety is a situation-specific personality trait generally regarded as having two psychological components: worry and emotional stimulation [5]. Test anxiety is considered as a broader “evaluation anxiety” construct and is composed of cognitive, emotional, behavioural, and bodily responses that are associated with concerns about potential negative outcomes or failure when on evaluative situations [1, 6–8]. Test anxiety is a crucial factor in determining academic outcomes, and it may lead to poor cognitive performance, academic underachievement and/or psychological distress [8].

Research on this topic is not new: the first studies on this subject date back to 1914, with the concept arising in 1952, when Text Anxiety Questionnaire was published by Mandler and Sarason [9]. In Liebert and Morri’s [10] early designation, test anxiety was analyzed as a bi-dimensional construct involving two components: worry and emotionality. Worry reflects the cognitive aspect of test anxiety and refers to concerns relating to performance during the exams, while emotionality encompasses students’ physical reactions experienced during the testing situation. Perhaps the most important contributions to test anxiety research were the distinction between anxiety as a temporary state and as a personality trait [5, 9] and the distinction between two basic dimensions in anxiety: worry and emotionality [5, 6, 9]. Later, in the 80’s, an influential theoretical shift into a multidimensional view of test anxiety emerged. Sarason and Wine postulated that test anxiety is a complex phenomenon that consists of cognitive, emotional, behavioral and bodily discriminable components [11, 12].

Considering test anxiety as a multidimensional construct, Sarason [11] established a four-factor Reactions to Test Scale (RTT) to assess this matter. Later, Benson [13] developed a shorter scale where they combined the RTT scale [11] and the Text Anxiety Inventory [14], and removed items that were redundant or incapable of loading substantially in any factor, creating, therefore, a scale that combined the strengths of both tools with a total of 18 items. They then pursued to enhance the precision of the scale by adding new items, especially in the Bodily symptoms component. The outcome was the 20-item RTT scale [6, 15].

In this work, we aimed to answer the call of Benson and Bandalos [13] for more validation studies of RTT 20-item with other populations to analyze the stability and generalization of the first and second-order factor models of RTT 20-item. Overall, there were three goals to the present work: (1) to understand the occurrence of test anxiety in a sample of Portuguese medical students, (2) determine if the validity and reliability of the RTT could be replicated in a Portuguese sample and (3) obtain data regarding the convergent validity of the RTT and STAI questionnaires. The tested hypothesis was that the RTT scale is a valid and reliable way to measure test anxiety in Portuguese medical students.

## Methods

### Sample

Data was collected from pre-clinical (the first three) and clinical (the last three) years of medical college students from the School of Medicine of the University of Minho (UM) and the Nova Medical School in Lisbon (NMS/UNL). Regarding missing values, a Little's MCAR test was performed to understand whether missing values were randomly distributed. Considering that the test revealed no statistical significance, we assume that the data is missing completely at random and, therefore, we proceeded to replace missing values by the variable median. The final sample comprised 672 medical students (553 in pre-clinical phase and 119 at the clinical phase). Age ranged from 17 to 39, with a mean of 20.6 (SD = 2.75. Of these, five hundred and eleven were females (76%), and one hundred and sixty-one were males (24%).Of the 672 students in this sample, 393 belong to UM and 279 to NMS/UNL. At NMS/UNL, 21.9% of the students are male and the rest are female (n = 218). This tendency remains when we refer to UM, where 25.4% of the students are male and 74.6% are female. The average age in both universities, 19.35 (SD = 2.28) at NMS/UNL and 21.43 (SD = 2.73) at UM. The higher mean age at UM may be explained by the fact that between the two universities, only UM has students in the last 3 years of the course, i.e., in the clinical phase (n = 119).

EFA was performed on a randomly selected half of the data to examine the factor structure of the scale. A CFA was conducted in the other half of the sample. The gender distribution of the EFA sample is 24.7% male (n = 83) and 75.3% female (n = 253). The sample has an average age of 20.4 years (SD = 2.76), with 188 students belonging to UM, and the rest being students from NMS/UNL (n = 148). Considering the clinical phase, 83.9% of the participants were in the preclinical phase (first 3 years) and the remaining were in the last 3 years of the academic pathway. For the CFA sample, the mean age was 20.67 years (SD = 2.74), with 23.2% being male and 76.8 female (n = 78 and 258, respectively) 0.188 students were students from UM and 148 from NMS/UNL and about 80.7% of them were in the preclinical phase (first 3 years of the medical degree).

## Measures

### RTT Scale

Given the complexity of the test anxiety phenomenon, various instruments have been developed for its determination and analysis. The Reaction to Tests Scale (RTT) (see additonal files) is a measure of test anxiety based on the interference model proposed by Sarason [11] and it represents the first shift to a multidimensional view of test anxiety. The RTT evaluates four dimensions of test anxiety: (a) tension, (b) worry, (c) test-irrelevant thinking and (d) bodily symptoms. Specifically, the (a) tension assesses feelings of muscle tension; (b) worry evaluates the presence of distracting worrying thoughts related to test performance; (c) test-irrelevant thinking factor contains items that measure the frequency and intensity of thought that are irrelevant to the testing situation, and (d) bodily symptoms includes physiological symptoms of anxiety. Meanwhile, based on the theoretical four-factor dimensionality proposed by Sarason [11], Benson and Bandalos [13] developed a shorter 20-item scale revision by reducing redundant items of the 40-items RTT. Participants are asked to rate each item on a four-point Likert format scale (*1* = *not all typical of me, 2* = *only somewhat typical of me, 3* = *quite typical of me and 4* = *very typical of me*), except item 20 that is reverse coded. Five items measure tension, six evaluates worry, five measure test-irrelevant thinking and four measure bodily symptoms. This shorter version presented high reliability, ranging from 0.68 for bodily symptoms, 0.85 for test-irrelevant thinking, 0.82 for worry, 0.91 for tension and 0.90 for the total scale.

### STAI-Y

The STAI-Y [16] is a measure composed by two subscales with 20 items allocated to each of them. The State Anxiety subscale (S – Anxiety) evaluates the current state of anxiety asking how individuals feel “right now”. The trait anxiety subscale (T – Anxiety) assesses relatively stable aspects of “anxiety proneness”. Each item is scored on a scale of 1 to 4, based on the intensity and frequency. Range of scores of each subscale is 20–80, the higher score representing greater anxiety.

### Procedure

The study was conducted in two moments: (1) translation of the English version into Portuguese and (2) validation of the Portuguese version. The translation process of RTT was conducted according to the following steps: (a) translation of the English version into Portuguese by one person without prior knowledge of the subject and two people with knowledge in the area; (b) direct comparison of the translated versions and synthesis of a single Portuguese version of RTT, after solving discrepancies through consensus; (c) back-translation and (d) pilot test of the pre-final Portuguese version on a randomly selected sample of medical students (n = 67). After modifications in RTT, the final version (additional file 1) was applied to the participants in the validation phase of the study. No changes were made to the scoring system and the rating criteria of the original instrument.

### Data analysis

Data regarding the sample and RTT psychometric characteristics was analysed using IBM SPSS version 26. RStudio Version 1.2.5042 was used for reliability analysis, EFA, and CFA. Descriptive statistics of the RTT scale included the mean score, standard deviation, skewness (Sk) and kurtosis (Ku). Values higher than three for Sk and 10 for Ku were considered as severe violations of normal distribution of the items (Kline, 2011). The sample was randomly split in half to allow for independent EFA and to test the best fit model—CFA. The factorial structure was explored in RStudio by performing an EFA using the GPArotation package [18] and a CFA using the Lavaan package [19]. Parallel analysis was used to determine the number of extracted factors. CFA confirmed the best fit model, and it was also used to test both the first-order factor structure (four subscales) and second-order factor structure (four subscales related to a general factor, Test Anxiety). As in the study by Benson and El-Zahhar [6], the present study sought to see if the correlation between factors could translate into a more general construct—test anxiety.

In order to evaluate model fit, chi-square by degrees of freedom ratio (**χ**^2^/df), Comparative Fit Index (CFI), Tucker-Lewis index (TLI), Root Mean Square Error of Approximation (RMSEA) and Akaike Information Criterion (AIC) were used. The model was considered to have an acceptable fit if the value of **χ**^2^/df was less than five, RMSEA < 0.08 [20], and if CFI and TLI > 0.9 [20]. For AIC, the model with the lowest values fits the data better [21].

Modification Indices were also analyzed to identify correlations among errors, considering values above 11. From the values above this limit, the one that had the most significant value was added. Internal consistency of the RTT was assessed through Cronbach’s alpha (α) and McDonald’s Omega (ω) for the total scale and each of the four subscales. Composite Reliability (CR) and Average Variance Extracted (AVE) were also computed, considering the criteria of ≥ 0.5 as acceptable value [21]. Convergent validity was assessed by testing correlation matrix between the RTT and STAI-Y. Both Scales were implemented simultaneously to UM students.

Gender and curriculum phase differences were studied by applying an independent t-test to the RTT Total scale (test anxiety), and multivariate analysis of variance (MANOVA) to RTT subscales. The effect size, along with the 95% confidence interval for gender and pre-clinical/clinical years differences in each RTT subscale and total scale was also calculated, considering benchmarks of effect sizes proposed by Cohen (small: d = 0.2; medium: d = 0.5; large: d = 0.8) [22, 23].

To explore the comparability of measured attributes across subgroups of respondents [24], measurement invariance was tested for gender and curriculum phase. Five nested models with gradually constricted parameters were tested: Model 1 tested for Configural invariance (basic model structure), Model 2 tested for Metric invariance (same loadings across groups), Model 3 for Scalar invariance (constrained factor loadings and item intercepts) and Model 4 for Residual invariance (same measurement errors) [25]. The differences between nested models regarding CFI and RMSEA indices were considered acceptable for the following values: ΔCFI ≤  − 0.02, ΔRMSEA ≤ 0.03, for tests of factor loading invariance and ΔCFI ≤ − 0.01 and RMSEA ≤ 0.01 for testing scalar invariance [26].

## Results

### Descriptive statistics

Descriptive statistics for RTT items are presented in Table 1. Items’ sensitivity was assessed through Sk and Ku analysis, with values higher than 3 and 10, respectively, indicating severe deviance from normal distribution of the items [17]. All items show acceptable Skewness (ranging from − 0.86 to 2.10) and kurtosis (ranging from − 1.13 to 3.78).

**Table 1:** Descriptive statistics of the RTT items

Item	Median	Mean	Standard deviation	Skewness	Kurtosis
RTT1	2	1.96	0.89	0.62	− 0.42
RTT2	3	3.28	0.82	− 0.86	− 0.14
RTT3	2	2.39	0.98	0.26	− 0.94
RTT4	3	3.25	0.82	− 0.77	− 0.39
RTT5	2	2.08	0.96	0.54	− 0.67
RTT6	3	2.99	0.93	− 0.48	− 0.80
RTT7	1	1.56	0.86	1.48	1.28
RTT8	1	1.75	0.93	1.04	0.070
RTT9	3	2.68	0.95	− 0.09	− 0.98
RTT10	2	2.21	0.90	0.39	− 0.59
RTT11	2	1.94	0.93	0.66	− 0.52
RTT12	1	1.37	0.73	2.10	3.78
RTT13	2	2.23	1.06	0.34	− 1.13
RTT14	2	1.88	0.97	0.81	− 0.44
RTT15	3	2.91	1.00	− 0.40	− 1.04
RTT16	2	1.93	1.07	0.74	− 0.83
RTT17	3	2.75	0.98	− 0.20	− 1.03
RTT18	2	2.41	1.06	0.14	− 1.02
RTT19	1	1.71	0.85	0.98	0.10
RTT20	3	2.88	0.93	− 0.31	− 0.88

### Exploratory factor analysis

**Fig. 1:**
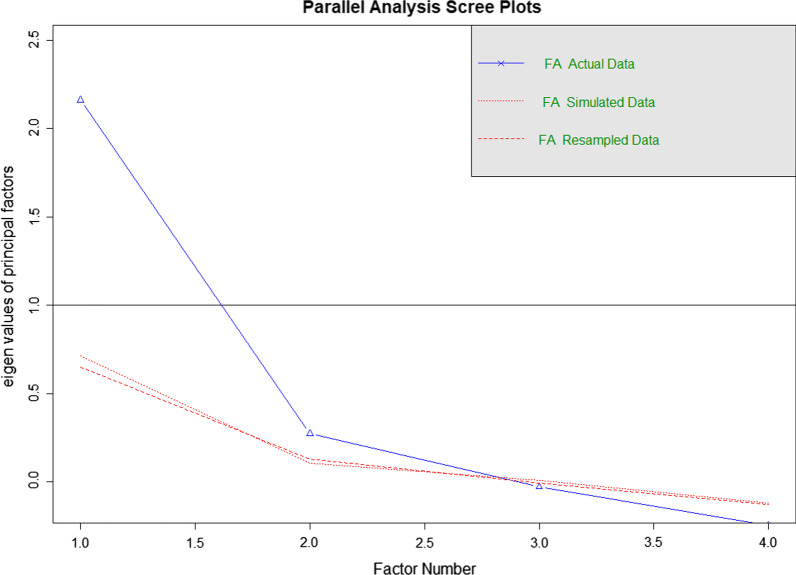
Parallel analysis

The dataset was split into two random samples. EFA was performed in 336 randomly selected individuals. To estimate the number of factors to retain, a parallel analysis was performed. Parallel analysis estimated four factors, as seen in Fig. 1.

Successively, a four-factor solution was inspected with an EFA with a principal axis factor analysis using a *promax* rotation, with loadings below 0.30 suppressed. EFA revealed a four-factor structure, similar to the one reported by Benson and Bandalos [13]. The four factors explained 45.0% of the variance. Standardized factorial weights and individual item’s reliability for the model are presented in Table 2. The factors were construed as Test Irrelevant Thinking, Tension, Worry and Bodily Symptoms.

**Table 2:** Exploratory Factor analysis: factor patter matrix for the Portuguese RTT scale

Item	Test irrelevant thinking	Tension	Worry	Bodily Symptoms
RTT1				0.33
RTT2		0.88		
RTT3			0.67	
RTT4		0.80		
RTT5	0.76			
RTT6			0.40	
RTT7			0.32	
RTT8				0.61
RTT9		0.35		
RTT10			0.72	
RTT11	0.94			
RTT12	0.60			
RTT13				0.43
RTT14	0.73			
RTT15		0.79		
RTT16				0.54
RTT17		0.57		
RTT18			0.68	
RTT19	0.65			
RTT20			0.30	

### Confirmatory factor analysis

CFA confirmed the other half of the initial sample (N = 336), which supported the four-factor structure for first-order factor, with almost all items loading substantially on hypothesized factors. Loadings ranged from 0.22 to 0.85. Only item 20 presented a value below 0.40. (Cf. Table 3).

**Table 3:** Confirmatory factor analysis: factor pattern matrix for the Portuguese RTT scale

Item	Test irrelevant thinking	Tension	Worry	Bodily symptoms
RTT1				0.38
RTT2		0.58		
RTT3			0.77	
RTT4		0.60		
RTT5	0.73			
RTT6			0.56	
RTT7			0.50	
RTT8				0.63
RTT9		0.72		
RTT10			0.70	
RTT11	0.80			
RTT12	0.56			
RTT13				0.82
RTT14	0.67			
RTT15		0.85		
RTT16				0.49
RTT17		0.80		
RTT18			0.82	
RTT19	0.60			
RTT20			0.22	

**Fig. 2:**
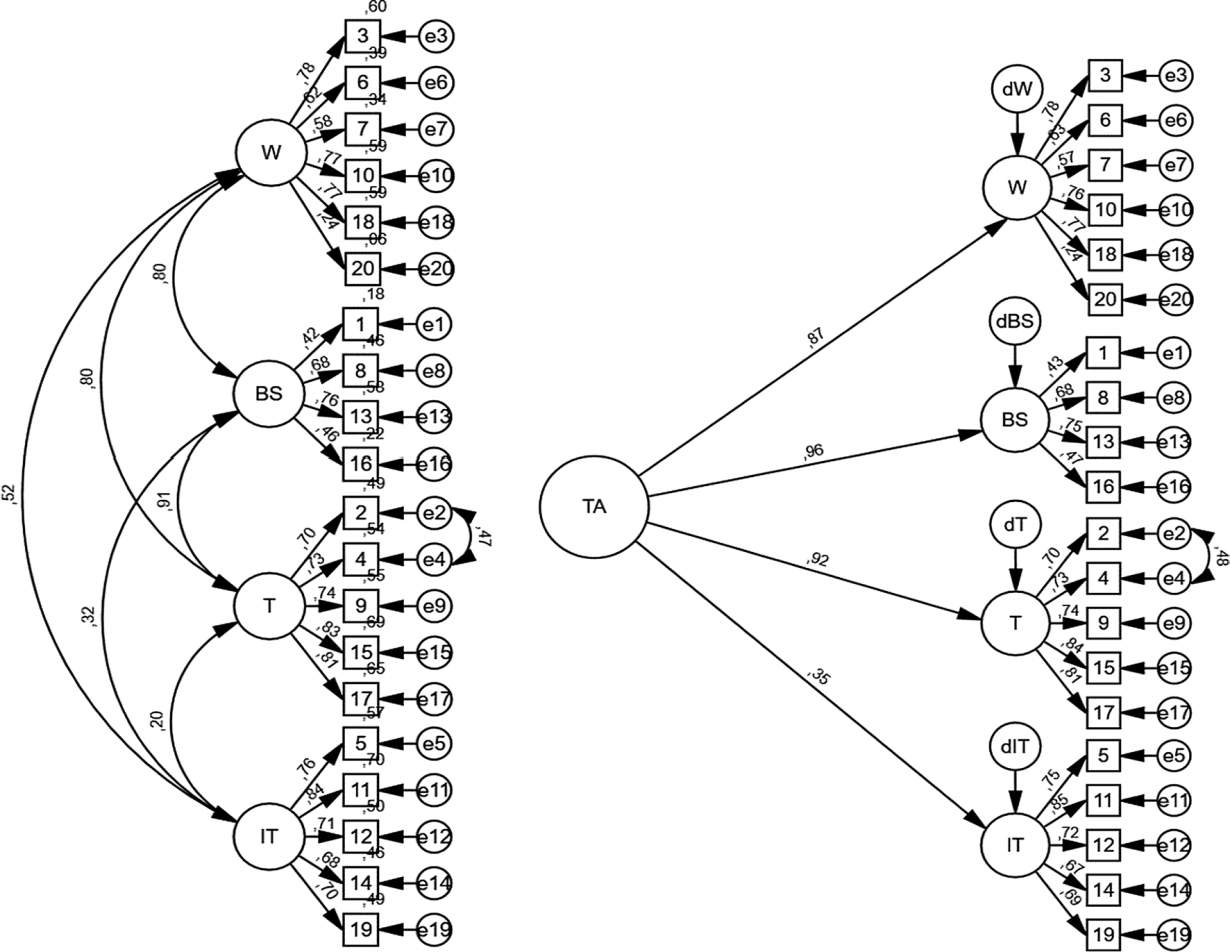
Modified first and second order models

Fit indices suggested that the model provided a good fit for the data, as seen in Table 4. The first-order model revealed satisfactory fit indices (**χ**^2^/df = 2.9, CFI = 0.90, RMSEA = 0.075, SRMR = 0.059), although TLI did not meet the previously stipulated criteria for acceptable fit (TLI = 0.89). The Modification index revealed a correlation between errors in the tension subscale, which were added to the previously computed model, resulting in a new modified model with satisfactory fit indices (Cf. Figure 2).

**Table 4:** Goodness of fit indexes for Portuguese RTT scale

	χ^2^	df	χ^2^/df	TLI	CFI	RMSEA	AIC	SRMR
1st order model	473	164	2.91	0.89	0.903	0.075	15,397	0.059
Modified 1st order model	407	163	2.50	0.91	0.923	0.067	15,333	0.056
2nd order model	534	166	3.21	0.87	0.884	0.081	15,454	0.076
Modified 2nd order model	464	165	2.82	0.89	0.91	0.074	15,388	0.073

Regarding the second-order latent factor model (test anxiety), only **χ**^2^/df reached acceptable values. The correlation between errors was added (e2 and e4) (Cf. Fig. 2) improving overall model fit, except for TLI, whose value did not reach the target level (TLI = 0.89). Concerning AIC values and goodness of fit indices, the first-order factor model with correlated errors presented the lowest value for AIC and best-fit indices.

### Reliability

In the present study, the RTT scale demonstrated high internal consistency with a Cronbach’s α of 0.90 for the total scale (Cf. Table 5). Tension, Worry, and Test Irrelevant thinking subscales evidenced values above 0.7 (Tension: α = 0.88; Worry: α = 0.79; Test Irrelevant thinking: α = 0.85), except the Bodily Symptoms subscale (α = 0.67). Composite reliability values were adequate for all subscales, except bodily symptoms (which revealed, nevertheless, an acceptable value (CR = 0.68)). Whilst AVE being satisfactory for Tension and Test Irrelevant Thinking, it fails to reach the expected values in tension and bodily symptoms subscales and the total scale. Similarly to Cronbach’s α, McDonald’s ω values were acceptable for the total scale and all subscales, except Bodily Symptoms.

**Table 5:** Reliability values for the subscales and total scale

	Worry	Tension	Test irrelevant thinking	Bodily symptoms	Test anxiety
Cronbach’s alpha	0.79	0.88	0.85	0.67	0.90
AVE	0.44	0.60	0.55	0.36	0.49
CR	0.81	0.86	0.86	0.68	0.92
McDonald’s omega	0.81	0.89	0.86	0.68	0.90

### Correlation between subscales

**Fig. 3:**
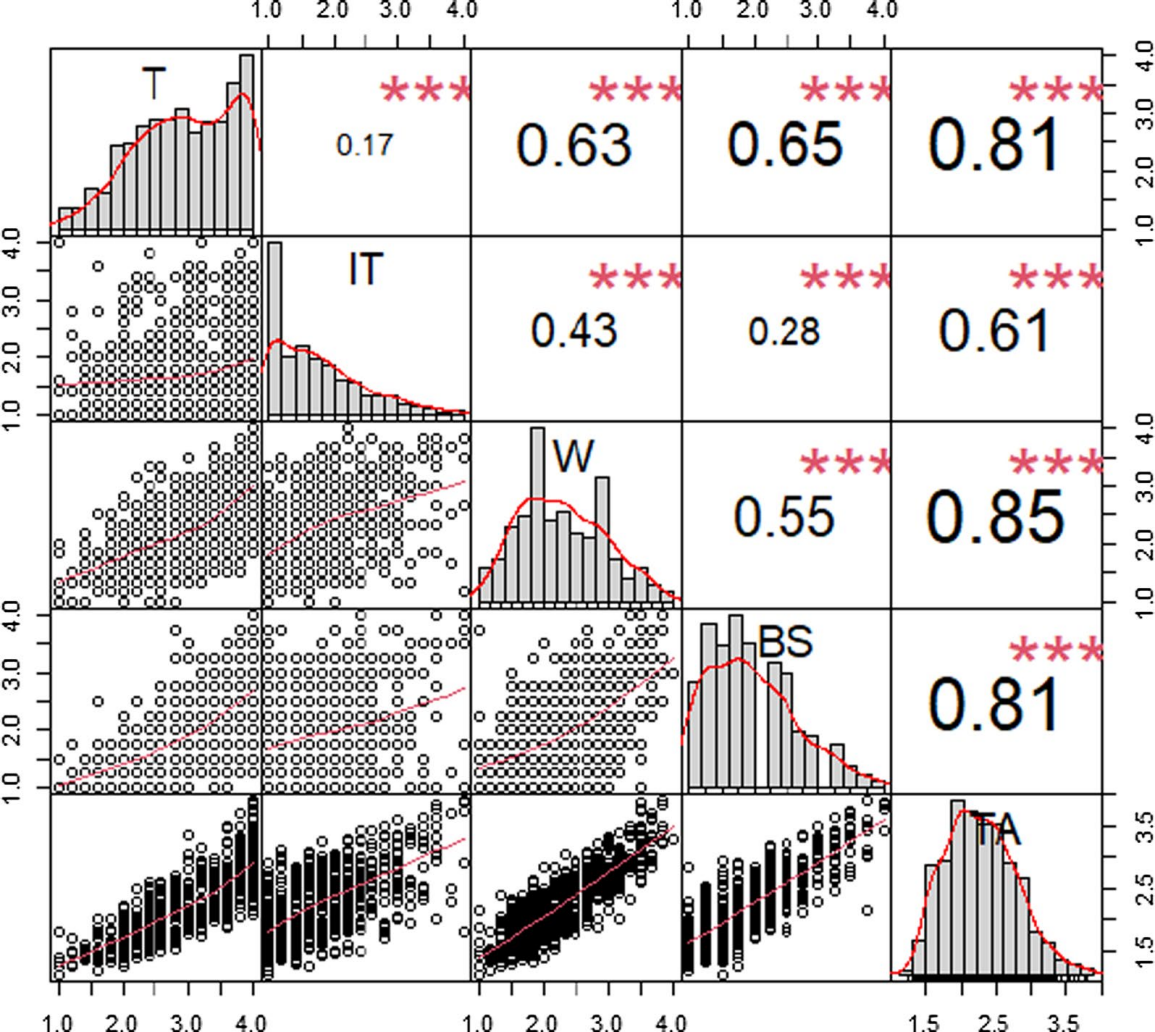
Correlation matrix between subscales and RTT scale. *T* tension, *W* worry, *BS* bodily symptoms, *IT* test irrelevant thinking, *TA* test anxiety

Figure 3 shows the correlation matrix between the subscales and the total scale.

Reported correlations between subscales Worry (W), Tension (T) and Bodily Symptoms (BS) range between 0.55 and 0.65, while those between Test Irrelevant Thinking (IT) and other factors were much lower, ranging from 0.17 and 0.43. For the correlation between subscales and total scale (TA), all values presented satisfactory correlations, with the lowest being for Test Irrelevant Thinking.

### Convergent validity

**Fig. 4:**
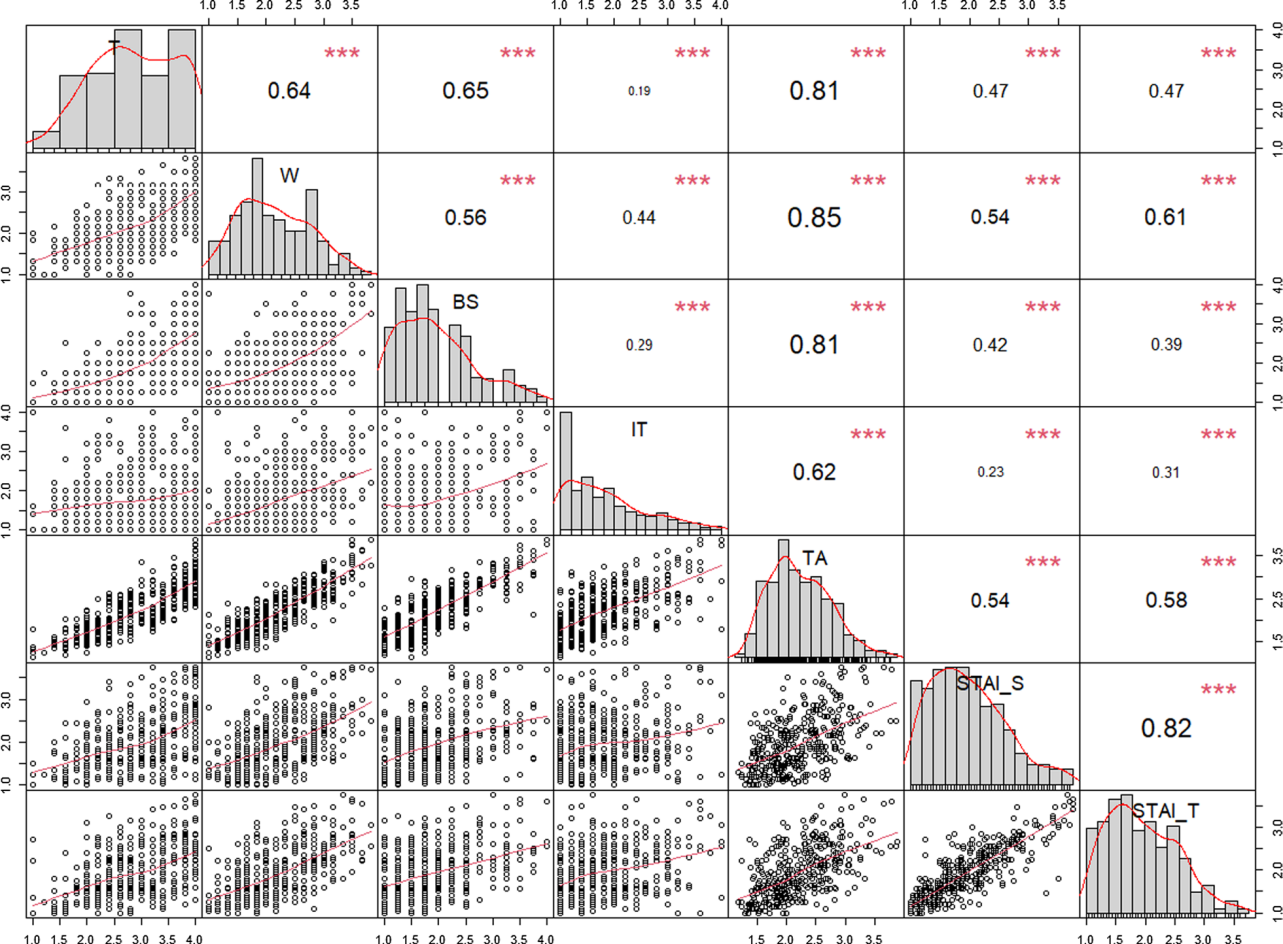
Correlation matrix between subscales, RTT scale and STAI. *T* tension, *W* worry, *BS* bodily symptoms, *IT* test irrelevant thinking, *TA* test anxiety, *STAI-S* state anxiety, *STAI-T* Trait anxiety

The RTT intercorrelations with STAI Inventory for the UM students (n = 393) are summarized in Fig. 4. Concerning state anxiety subscales, significantly and positively correlations with RTT subscales and total score were found. All the RTT subscales and total score are significantly positively associated with trait anxiety subscales, thus pointing to its convergent validity. The positive correlations between the measures are expected, as reported in Bados et al. [15].

### Measurement invariance

For Measurement invariance testing, a series of multi-groups CFA were conducted between groups: curriculum phase (pre-clinical vs clinical) and gender (male vs female) (Cf. Table 6). Regarding the second order CFA modified model, comparisons between gender show that configural invariance was supported, as it revealed good indexes of fit (CFI = 0.90, RMSEA = 0.075, AIC = 15,406, BIC = 15,902), meaning that configural invariance is maintained. We also found support for metric invariance (ΔCFI = 0.001, ΔRMSEA = 0.002, *p* = 0.595). Concerning Scalar invariance, the model is acceptable, as it fits in the suitable ranges (ΔCFI = 0.002, ΔRMSEA = 0.002, *p* = 0.838). Although strict invariance is supported by almost all fit measures for gender (ΔCFI = 0.01, ΔRMSEA = 0.0019), the *p* value is < 0.001, which may suggest that it does not fit our data particularly well.

**Table 6:** Measurement invariance for the CFA sample

Model	χ^2^	df	CFI	AIC	BIC	RMSEA	Δχ^2^	*p* value	ΔCFI	ΔRMSEA
*First-order CFA*										
Gender										
Configural invariance	583.19	326	.918	15,354	15,866	.069	–	–	–	–
Metric invariance	593.96	342	.920	15,333	15,783	.066	10.769	.820	.002	.002
Scalar invariance	603.49	358	.922	15,310	15,700	.064	9.522	.890	.002	.002
Strict invariance	654.82	378	.912	15,322	15,635	.066	51.333	< .001	.010	.002
Curriculum phase										
Configural invariance	583.67	326	.920	15,380	15,892	.069	–	–	–	–
Metric invariance	599.97	342	.920	15,365	15,815	.067	16.308	.430	.0	.002
Scalar invariance	631.78	358	.915	15,364	15,754	.067	31.741	.011	.005	0
Strict invariance	657.37	378	.913	15,350	15,663	.066	25.657	.177	.002	.001
Second-order CFA										
Gender										
Configural invariance	642.67	330	.901	15,406	15,902	.075	–	–	–	–
Metric invariance	659.59	349	.902	15,385	15,808	.073	16.92	.5952	.001	.002
Scalar invariance	669.59	364	.903	15,364	15,731	.071	9.702	.838	.002	.002
Strict invariance	719.34	384	.894	15,374	15,664	.072	50.04	< .001	.010	.001
Curriculum phase										
Configural invariance	646.2	330	.901	15,435	15,931	.076	–	–	–	–
Metric invariance	662.49	349	.902	15,413	15,837	.073	16.292	.637	.001	.002
Scalar invariance	694.79	364	.897	15,416	15,782	.074	32.302	.0058	.005	0
Strict invariance	718.73	384	.896	15,400	15,690	.072	23.936	.245	.001	.002

Measurement invariance was also tested for Phase (preclinical vs clinical). Table 6 shows that configural invariance is supported across both phases: CFI = 0.901, RMSEA = 0.076, AIC = 15,435, BIC = 15,931. Metric invariance was also supported, as all fit measures were within range (ΔCFI = 0.001, ΔRMSEA = 0.002, *p* = 0.637). Concerning scalar invariance, the model is upheld in almost all fit measures for gender (ΔCFI = 0.01, ΔRMSEA = 0.0019), except for the *p* value (< 0.001). Strict invariance was supported by all fit measures.

The same models were applied to the total sample (n = 672) (Cf. Table 7). Regarding the second-order CFA modified model for the total sample, only strict invariance for gender was not supported (ΔCFI = 0.010, ΔRMSEA = 0.001, *p* value < 0.001).

**Table 7:** Measurement invariance for the total sample

Model	χ^2^	df	CFI	AIC	BIC	RMSEA	Δχ^2^	*p* value	ΔCFI	ΔRMSEA
*First-order CFA*										
Gender										
Configural invariance	680.89	326	.939	30,557	31,162	.057	–	–	–	–
Metric invariance	694.84	342	.939	30,539	31,071	.055	13.946	.603	.000	.002
Scalar invariance	713.29	358	.939	30,526	30,986	.054	18.452	.298	.000	.001
Strict invariance	788.93	378	.929	30,561	30,931	.057	75.634	< .001	.010	.003
Curriculum phase										
Configural invariance	670.77	326	.942	30,645	31,249	.056	–	–	–	–
Metric invariance	690.73	342	.942	30,633	31,165	.055	19.954	.222	.001	.001
Scalar invariance	721.03	358	.939	30,631	31,091	.055	30.303	.017	.002	.000
Strict invariance	748.86	378	.938	30,619	30,989	.054	27.829	.114	.001	.001
*Second-order CFA*										
Gender										
Configural invariance	781.37	330	.922	30,650	31,236	.064	–	–	–	–
Metric invariance	796.78	349	.923	30,627	31,128	.062	15.414	.696	.001	.002
Scalar invariance	815.27	364	.922	30,616	31,049	.061	18.483	.238	.001	.001
Strict invariance	891.03	384	.913	30,651	30,994	.063	75.766	< .001	.010	.002
Curriculum phase										
Configural invariance	775.98	330	.925	30,742	31,328	.063	–	–	–	–
Metric invariance	795.52	349	.925	30,724	31,224	.062	19.534	.423	.000	.002
Scalar invariance	826.49	364	.922	30,725	31,158	.061	30.968	.009	.003	.000
Strict invariance	853.06	384	.921	30,711	31,054	.060	26.571	.148	.001	.001

### Gender and year of medical training comparison

Gender and phase differences for the total scale were analyzed through an independent samples Student’s T-test. Concerning gender, significant statistical differences with medium effect sizes were found in the total scale (*t* (670) = − 5.1; *p* < 0.001, *d* = 0.46(0.28–0.64)) with female medical students reporting higher scores than male students. No statistical differences were found concerning phase (*t* (670) = 1.3; *p* = 0.189, *d* = 0.13(0.065–0.331)).

A MANOVA was conducted to test the hypothesis that there would be differences between gender and phase at a subscale level. For gender, we obtained significant results: the Tension, Worry and Bodily Symptoms subscales were significantly different in terms of gender (F (1, 670) = 42.4, *p* < 0.001; F (1, 670) = 28.1, *p* < 0.001 and F (1, 670) = 12.9, *p* < 0.001). Only Test Irrelevant thinking showed no differences for gender (F (1, 670) = 0.007, *p* = 0.934). Concerning the curriculum phase, with a MANOVA analysis no significant differences were found.

## Discussion

The three main objectives of this study were to understand the occurrence of test anxiety in a sample of Portuguese medical students, to determine if the validity and reliability of the RTT could be replicated in a Portuguese sample and to obtain data regarding the convergent validity of the RTT and STAI questionnaires. The EFA reinforced the expected 4-factor structure. CFA validated the first and second-order factor model [13]. Nevertheless, even though both demonstrated good model fit, the TLI of the modified second-order factor was slightly below the acceptable values (TLI = 0.89). In terms of internal consistency and composite reliability, the results suggest that the sensitivity, construct validity and reliability of the RTT scale were acceptable. Only the Bodily Symptoms subscale presented a Cronbach’s α value below 0.70, but in an acceptable level (≥ 0.60), which can be explained by the fact that Cronbach’s alpha is influenced by the number of items [27] and this subscale is the only with 4 items. These results are similar to those reported by Benson et al. [13] and Bados et al. [14]. For AVE, the Worry and Bodily Symptoms subscales and the total Test Anxiety scale presented values below 0.5. This can be because the worry subscale has one item (20-*Após um teste, digo para mim mesmo “Acabou e eu fiz o melhor que pude”*) that has loading inferior to 0.3. However, since the Worry subscale and the total Test Anxiety scale CR value were above 0.7, this value is acceptable (Fornell et al., 1981). In contrast, the Bodily Symptoms subscale reached neither the AVE nor the CR value. The scale has only four items, one of which (item 1-*A minha boca fica seca durante um exame*) has a low loading value (0.38), which may explain this result.

Concerning convergent validity, the trait anxiety subscales for the UM students presented significantly and positively correlations with RTT subscales and total score, evidencing the expected convergent validity. Regarding gender, female medical students showed higher test anxiety scores compared to male students. There were no differences relating to curriculum phase in any of the subscales or total scale. Finally, as expected, support for metric invariance was found across different groups (gender and phase). Although strict invariance for gender was supported in almost all fit measures, the *p* value was < 0.001, which may indicate variance. Concerning phase, although the *p* value did not allow to prove the thesis of scalar invariance, the rest of the indices contradict this conclusion. However, chi-square statistic often has higher power to detect minor model misspecification with larger sample sizes, and since all the other parameters are according to the stipulated values, we can accept invariance [29].

This study allowed us to understand the test anxiety patterns of medical students at two Portuguese universities. Additionally, the validation of this scale will allow an increase in the standardization of results among countries where the scale is already validated. A potential limitation of our study is the fact that participants were only from one university course (medicine), which should be considered when generalizing results. Second, our study has a cross-sectional design and does not allow to analyze the stability of the RTT 20-item version over time. Third, the sample is mainly composed of female students and pre-clinical students, which limits the measurement invariance across gender and curriculum phase.

## Conclusions

To the best of our knowledge, our study is the first attempt to validate the RTT 20-item version in a sample of Portuguese medical students. Additionally, it responds to the call of Benson et al. [13] for validation studies of RTT 20-item with other populations to analyze the stability and generalization of the first and second-order factor models of the RTT 20-item version. The results support the validity and reliability of the Portuguese RTT 20-item among medical students and confirm the factor structure of the four-factor model (first order model) and the second order factor model. Given the challenges in applying test anxiety instruments across various cultures, the present study is a preliminary indicator that the RTT scale may prove a useful cross-cultural instrument.

## Supplementary Information

**Additional file 1**. The Portuguese and the English originals versions of the of Reaction to Test Scale.

## Data Availability

The datasets generated during and/or analyzed during the current study are not publicly available due confidentiality issues but are available from the corresponding author on reasonable request.

## Abbreviations

AIC: Akaike Information Criterion
AVE: Average Variance Extracted
CFA: Confirmatory Factor Analysis
CFI: Comparative Fit Index
CR: Composite reliability
EFA: Exploratory Factor Analysis
Ku: Kurtosis
NMS/UNL: Nova Medical School in Lisbon
RMSEA: Root Mean Square Error of Approximation
RTT: Reactions to tests Scale
Sk: Skewness
STAI-Y: State-Trait Anxiety Inventory
TLI: Tucker-Lewis index
UM: University of Minho
