# Supplementary material for: Applicability and validation of the Reaction to Tests Scale (RTT) in a sample of Portuguese medical students
Source: BMC Psychol. 2021 Oct 27;9:166. doi: 10.1186/s40359-021-00656-w (PMC8555089; doi:10.1186/s40359-021-00656-w)
Supplement: Supplementary file 1 — Additional file 1. The Portuguese and the English originals versions of the of Reaction to Test Scale. [file 40359_2021_656_MOESM1_ESM.docx]

**Applicability and Validation of the Reaction to Tests Scale (RTT) in a sample of Portuguese medical students**

Daniela S. M. Pereira (1,2,3) *, Ana Mónica Pereira (1,2) *, Teresa Costa Castanho (1,2), Gabriela A. Silva (4,5), Filipe Falcão (1,2,3), Patrício Costa (1,2), José Miguel Pêgo (1,2,3)

1. Life and Health Sciences Research Institute (ICVS), School of Medicine, University of Minho, Largo do Paço, 4700-000 Braga, Portugal

2. ICVS/3B's, PT Government Associate Laboratory, Largo do Paço, 4700-000 Braga, Portugal

3. iCognitus4ALL – IT Solutions, 4710-057 Braga, Portugal

4. Nova Medical School, Campo dos Mártires da Pátria 130, 1169-056 Lisboa, Portugal

5. iNOVA4Health, CEDOC, Edifício CEDOC II, Rua Câmara Pestana 6, 1150-082 Lisboa, Portugal

* These authors equally contributed to this manuscript.

Correspondence should be addressed to José M. Pêgo School of Medicine, University of Minho, Campus de Gualtar, 4710-057, Braga, Portugal; telephone: +351 253 604 932; e-mail: [jmpego@med.uminho.pt](mailto:jmpego@med.uminho.pt)

**Appendix**

**Tradução da Escala de Reações face aos testes – Benson *et al*.(1992)**

|  | Nada normal | Um pouco normal | Bastante normal | Muito normal | Introduzir a pontuação para cada questão. | | | |
| --- | --- | --- | --- | --- | --- | --- | --- | --- |
|  |  |  |  |  | **T** | **P** | **PI** | **SC** |
| 1. A minha boca fica seca durante um exame. | 1 | 2 | 3 | 4 |  |  |  | 🞎 |
| 1. Sinto-me nervoso antes dos testes. | 1 | 2 | 3 | 4 | 🞎 |  |  |  |
| 1. Pensamentos sobre ter um mau desempenho interferem com a minha concentração durante os testes. | 1 | 2 | 3 | 4 |  | 🞎 |  |  |
| 1. Fico ansioso(a) com os testes. | 1 | 2 | 3 | 4 | 🞎 |  |  |  |
| 1. Durante os testes, acho que fico distraído(a) com pensamentos de eventos futuros. | 1 | 2 | 3 | 4 |  |  | 🞎 |  |
| 1. Perante um teste difícil, preocupo-me se irei passar. | 1 | 2 | 3 | 4 |  | 🞎 |  |  |
| 1. Enquanto estou a fazer um teste, encontro-me a pensar o quão brilhantes as outras pessoas são. | 1 | 2 | 3 | 4 |  | 🞎 |  |  |
| 1. Fico com dores de cabeça antes de um teste. | 1 | 2 | 3 | 4 |  |  |  | 🞎 |
| 1. Enquanto faço um teste, sinto-me tenso. | 1 | 2 | 3 | 4 | 🞎 |  |  |  |
| 1. Durante os testes penso o quão mau está a ser o meu desempenho/quão mal estou a fazer. | 1 | 2 | 3 | 4 |  | 🞎 |  |  |
| 1. Durante os testes encontro-me a pensar em coisas não relacionadas com o tema avaliado/com o material a ser testado. | 1 | 2 | 3 | 4 |  |  | 🞎 |  |
| 1. Algumas vezes tenho fantasias durante um teste. | 1 | 2 | 3 | 4 |  |  | 🞎 |  |
| 1. Às vezes fico a tremer (agitado) antes ou durante os testes. | 1 | 2 | 3 | 4 |  |  |  | 🞎 |
| 1. Enquanto estou a fazer um teste às vezes penso em estar noutro lugar. | 1 | 2 | 3 | 4 |  |  | 🞎 |  |
| 1. Sinto-me angustiado/aflito e inquieto antes de testes importantes. | 1 | 2 | 3 | 4 | 🞎 |  |  |  |
| 1. Sinto necessidade de ir à casa de banho mais vezes do que o habitual durante um teste. | 1 | 2 | 3 | 4 |  |  |  | 🞎 |
| 1. Eu tenho uma sensação desconfortável antes de um teste importante. | 1 | 2 | 3 | 4 | 🞎 |  |  |  |
| 1. O pensamento “O que acontece se eu falhar este teste?” passa pela minha cabeça durante os testes. | 1 | 2 | 3 | 4 |  | 🞎 |  |  |
| 1. Eu penso sobre acontecimentos atuais durante um teste. | 1 | 2 | 3 | 4 |  |  | 🞎 |  |
| 1. Após um teste, digo para mim mesmo “Acabou e eu fiz o melhor que pude”. | 1 | 2 | 3 | 4 |  | 🞎 |  |  |
|  |  |  |  | Totais: |  |  |  |  |

**RTT Scale (English Version) – Benson *et al*.(1992)**

|  | Almost Never | Somewhat Typical | Quite Typical | Almost Always | Enter Score for Each Question. | | | |
| --- | --- | --- | --- | --- | --- | --- | --- | --- |
|  |  |  |  |  | **T** | **W** | **IT** | **BS** |
| 1. My mouth feels dry during a test. | 1 | 2 | 3 | 4 |  |  |  | 🞎 |
| 1. I feel jittery before tests. | 1 | 2 | 3 | 4 | 🞎 |  |  |  |
| 1. Thoughts of doing poorly interfere with my   concentration during tests. | 1 | 2 | 3 | 4 |  | 🞎 |  |  |
| 1. I am anxious about tests. | 1 | 2 | 3 | 4 | 🞎 |  |  |  |
| 1. During tests, I find I am distracted by thoughts of upcoming events. | 1 | 2 | 3 | 4 |  |  | 🞎 |  |
| 1. During a difficult test, I worry whether I will pass it. | 1 | 2 | 3 | 4 |  | 🞎 |  |  |
| 1. While taking tests, I find myself thinking how much brighter the other people are. | 1 | 2 | 3 | 4 |  | 🞎 |  |  |
| 1. I get a headache before a test. | 1 | 2 | 3 | 4 |  |  |  | 🞎 |
| 1. While taking a test, I feel tense. | 1 | 2 | 3 | 4 | 🞎 |  |  |  |
| 1. During tests I think of how poorly I am doing. | 1 | 2 | 3 | 4 |  | 🞎 |  |  |
| 1. During tests I find myself thinking of things unrelated to the material being tested. | 1 | 2 | 3 | 4 |  |  | 🞎 |  |
| 1. I have fantasies a few times during a test. | 1 | 2 | 3 | 4 |  |  | 🞎 |  |
| 1. I sometimes find myself trembling before or during tests. | 1 | 2 | 3 | 4 |  |  |  | 🞎 |
| 1. While taking tests, I sometimes think about being somewhere else. | 1 | 2 | 3 | 4 |  |  | 🞎 |  |
| 1. I feel distressed and uneasy before important tests. | 1 | 2 | 3 | 4 | 🞎 |  |  |  |
| 1. I feel the need to go to the toilet more often than usual during a test. | 1 | 2 | 3 | 4 |  |  |  | 🞎 |
| 1. I have an uncomfortable feeling before an important test. | 1 | 2 | 3 | 4 | 🞎 |  |  |  |
| 1. The thought “What happens if I fail this test?” goes through my head during the tests. | 1 | 2 | 3 | 4 |  | 🞎 |  |  |
| 1. I think about current events during a test. | 1 | 2 | 3 | 4 |  |  | 🞎 |  |
| 1. After a test, I say to myself "It's over and I did the best I could". | 1 | 2 | 3 | 4 |  | 🞎 |  |  |
|  |  |  |  | Totals: |  |  |  |  |
